# Reducing the margin in prostate radiotherapy: optimizing radiotherapy with a general-purpose linear accelerator using an in-house position monitoring system

**DOI:** 10.3389/fonc.2023.1116999

**Published:** 2023-07-13

**Authors:** Sankar Arumugam, Karen Wong, Viet Do, Mark Sidhom

**Affiliations:** ^1^ Department of Medical Physics, Liverpool and Macarthur Cancer Therapy Centres and Ingham Institute, Sydney, NSW, Australia; ^2^ South Western Clinical School, University of New South Wales, Sydney, NSW, Australia; ^3^ Department of Radiation Oncology, Liverpool and Macarthur Cancer Therapy Centres, Sydney, NSW, Australia

**Keywords:** prostate radiotherapy, intrafraction motion, real-time position monitoring, CTV-PTV margin, treatment-related toxicity, quality of life

## Abstract

**Purpose:**

To study the feasibility of optimizing the Clinical Target Volume to Planning Target Volume (CTV-PTV) margin in prostate radiotherapy(RT) with a general-purpose linear accelerator using an in-house developed position monitoring system, SeedTracker.

**Methods:**

A cohort of 30 patients having definitive prostate radiotherapy treated within an ethics-approved prospective trial was considered for this study. The intrafraction prostate motion and the position deviations were measured using SeedTracker system during each treatment fraction. Using this data the CTV-PTV margin required to cover 90% of the patients with a minimum of 95% of the prescription dose to CTV was calculated using van Herk’s formula. The margin calculations were performed for treatment scenarios both with and without applying the position corrections for observed position deviations. The feasibility of margin reduction with real-time monitoring was studied by assessing the delivered dose that incorporates the actual target position during treatment delivery and comparing it with the planned dose. This assessment was performed for plans generated with reduced CTV-PTV margin in the range of 7mm-3mm.

**Results:**

With real-time monitoring and position corrections applied the margin of 2.0mm, 2.1mm and 2.1mm in LR, AP and SI directions were required to meet the criteria of 90% population to receive 95% of the dose prescription to CTV. Without position corrections applied for observed position deviations a margin of 3.1mm, 4.0mm and 3.0mm was required in LR, AP and SI directions to meet the same criteria. A mean ± SD reduction of 0.5 ± 1.8% and 3 ± 7% of V60 for the rectum and bladder can be achieved for every 1mm reduction of PTV margin. With position corrections applied, the CTV D99 can be delivered within -0.2 ± 0.3 Gy of the planned dose for plans with a 3mm margin. Without applying corrections for position deviations the CTV D99 was reduced by a maximum of 1.1 ± 1.1 Gy for the 3mm margin plan and there was a statistically significant difference between planned and delivered dose for 3mm and 4mm margin plans.

**Conclusion:**

This study demonstrates the feasibility of reducing the margin in prostate radiotherapy with SeedTracker system without compromising the dose delivery accuracy to CTV while reducing dose to critical structures.

## Introduction

Prostate cancer is the most commonly diagnosed cancer among men (1, 2). More than 60% of prostate cancer patients receive radiotherapy (RT) for the treatment of their disease (3). External beam radiotherapy (EBRT) is an effective treatment for prostate cancer. Radiotherapy planning and delivery techniques continuously evolve to improve the dose conformity to the intended treatment volume and reduce the dose to nearby Organs at Risk (OARs) such as the rectum and bladder. Studies have shown that these advancements resulted in reduced treatment-related toxicity after radiotherapy (4–6). Further, the advancements in image-guided radiotherapy (IGRT) enabled the accurate positioning of the target volume before the start of treatment. Gill et al (7) studied a large cohort of prostate cancer radiotherapy patients treated with and without IGRT. The cohort of patients included in their study are treated with the same dose prescription, treatment technique and treatment margin. Their analysis showed that the patients treated in the IGRT group had less urinary toxicity, diarrhea and fatigue in comparison to the non-IGRT group demonstrating the treatment quality improvement that can be achieved with IGRT.

Despite advancements in RT delivery, there remains a proportion of patients that develop acute and late gastrointestinal (GI) and genitourinary (GU) toxicity after prostate RT (8). This affects patients’ physiological and psychological well-being and results in poor quality of life after treatment (9). A Clinical Target Volume to Planning Target Volume (CTV-PTV) margin is used in prostate RT to account for target position uncertainties, and the larger this expansion there is a resulting in higher dose delivered to the rectum and bladder which contributes to the toxicity. The advancements in real-time target position monitoring during treatment delivery reduce the uncertainty of target position and unlock the possibility to reduce the magnitude of CTV-PTV margin thereby reducing the OARs dose. Several studies report real-time prostate position monitoring using implanted gold fiducial markers with megavoltage (MV) Electronic Portal Images (EPIs) (10, 11), implanted electromagnetic transponders (12, 13), intrafraction kilovoltage (kV) Cone Beam Computed Tomographic (CBCT) images (13, 14), kV stereoscopic or fluoroscopic images with implanted fiducials (15, 16) and trans abdominal or trans perineal ultrasound systems (17, 18).

The widespread application of prostate position monitoring in routine clinical practice is limited to prostate stereotactic body radiotherapy (SBRT) where a smaller magnitude of CTV-PTV margin is used to limit the dose to nearby OARs (17, 19, 20). One of the main reasons for this limited application is the requirement of dedicated additional position monitoring systems to enable real-time position monitoring in treatment with a general-purpose linear accelerator (linac). The feasibility of using the kV imaging system available in linacs to perform real-time monitoring for prostate SBRT has been reported (21–23). Our group developed such a system that enables real-time position monitoring in Elekta linacs with the XVI system (23–25). Recently, we completed a phase 1 trial (ACTRN12618001421224) that investigated the feasibility of using this system for conventionally fractionated prostate RT (26, 27). In this study, we investigate the feasibility of optimizing the CTV-PTV margin using the patient and real-time prostate position data collected in our phase-1 trial to explore the safe implementation of margin reduction in prostate RT in a general-purpose linac without the need for an additional position monitoring system.

## Methods

### Patient data

The patient data is sourced from an ethics-approved prospective trial (ACTRN12618001421224) that investigated the feasibility of using in-house developed position monitoring software, SeedTracker, for intrafraction prostate position monitoring and corrections in a general-purpose linear accelerator. A cohort of 30 prostate cancer patients with low-intermediate (15 patients) and high-risk (15 patients) categories was considered for this study. The patients implanted with intraprostatic gold fiducial markers were included in this trial with the exclusion of patients with hip prostheses.

### Real-time target position data

The real-time intrafraction prostate position data measured by the SeedTracker system was used for this study. In the original prospective trial, a cohort of 10 patients was treated with 5 mm, 4 mm and 3 mm position tolerance categories to study the acceptance of the system in the routine clinical environment. All the patients were positioned for treatment using pre-treatment Cone Beam Computed Tomography (CBCT) images acquired using Elekta XVI imaging system. The technical details of the SeedTracker system have been reported elsewhere (23–25). The patients were treated with either single or dual VMAT arc depending on the complexity of the plans. For the real-time position monitoring, x-ray images were acquired at a gantry angle spacing of 9° using 8 cm x 8 cm aperture. This imaging protocol resulted in the imaging dose of 0.9 mSv for the entire treatment course of 20 fractions (27). The mean (SD) beam- on time of patients treated within this study was 2.3(0.3)mins.

### PTV margin calculation

The required PTV margin with and without real-time position monitoring was calculated using van Herk’s non-linear margin recipe (28).


$$M=C_{1}\text{Σ}+C_{2}\left(\sqrt{\sigma^{2}+\sigma_{p}\;2}-\sigma_{p}\right)$$

Where Σ is the standard deviation of mean displacements (systematic error), σ is the standard deviation of the random variations and $\sigma_{p}$ (3.2 mm) is the width of the penumbra modeled by cumulative gaussian. $C_{1}$ is the confidence level corresponding to the percentage of the population and $C_{2}$ is the coefficient for dose level. The values of $C_{1}$ and $C_{2}$ were chosen to achieve 90% of the population having CTV covered by 95% of the prescription dose which correspond to 2.5 and 1.64 respectively.

### Radiotherapy plans with different PTV margins

**Figure 1:**
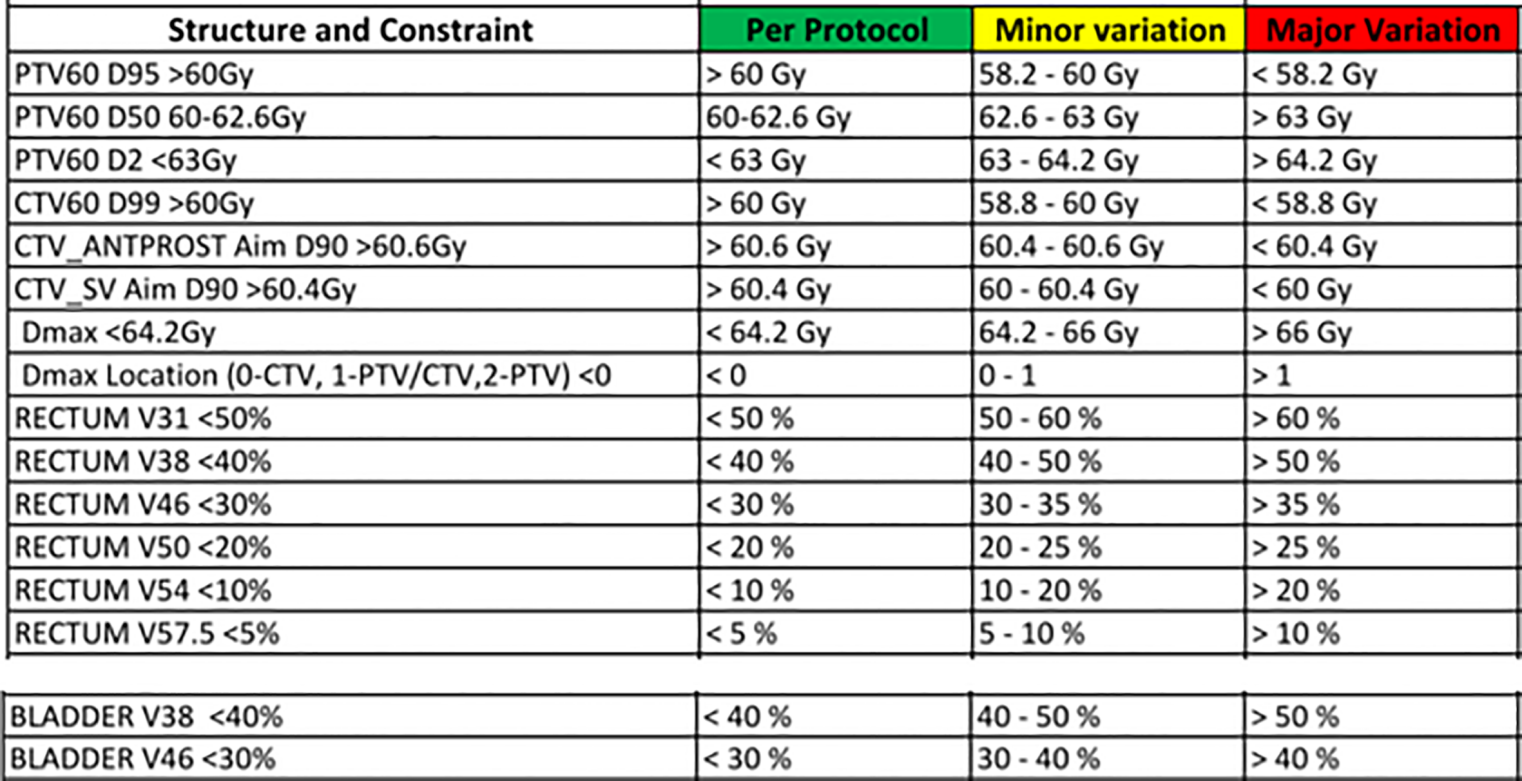
The departmental clinical goal protocol to evaluate the acceptability of prostate radiotherapy plans.

For this study, only prostate CTV was considered. The patients’ plans with pelvis node inclusion were replanned with prostate CTV only. Currently, we use 7 mm isotropic margins in our clinic to account for target position and geometrical uncertainties in prostate CTV during treatment delivery. To study the magnitude of dose reduction to OARs that can be achieved with reduced margins and the impact of prostate position deviations, additional treatment plans were generated with 6 mm, 5 mm, 4 mm and 3 mm margins. All the treatment plans were generated using a 6MV beam model for Elekat linac with Agility Multi Leaf Collimator in the Pinnacle treatment planning system (TPS). A dose prescription of 60 Gy in 20 fractions was used for all the plans and the clinical acceptability of the plans were assessed based on the departmental clinical dosimetric goals for prostate RT plan evaluation (**Figure 1**). To minimize the inter-planner subjectivity on the plan quality and maintain the consistency between the plans, all the plans were generated using the Autoplanning module in the Pinnacle TPS.

### Target volumes and OARs dose with reduced treatment margins

The CTV and PTV dose of plans generated with different PTV margins was compared using dose received by 99% of volume (D99) and dose received by 98% volume (D98) Dose Volume Histogram (DVH) metrics respectively. The reduction in dose to rectum and bladder that can be achieved with different CTV-PTV margins was compared using volume receiving 46 Gy (V46) and volume receiving 60 Gy (V60) DVH metrics. The statistical significance of dose difference between the plans generated with different PTV margins at the population level was studied using one-way Analysis of Variance (ANOVA) and Tukey’s honestly significant difference (HSD) test to the significance level of p<0.05.

### The delivered dose assessment

The dose delivered to target volumes and OARs was studied by incorporating the target positions determined by the SeedTracker system by the voxel-shift method (29–31). The dose delivered with position corrections applied to the observed position deviations (Corrected) was assessed by incorporating the residual position deviations below the action threshold to the 3D dose cube of the VMAT arc in each treatment fraction. The dose that would have been delivered without monitoring (Not corrected) was assessed by the following steps:

* In the treatment fractions where position deviations did not occur, the residual position errors were incorporated into the VMAT arcs as in the corrected scenario
* In the events where the position deviations occurred at the start of the treatment, the observed position deviation was incorporated into the whole treatment fraction
* In the events where the position deviations occurred during the delivery of the treatment, the residual error calculated up to the fraction of treatment delivery was incorporated for the 3D dose cube of control points (CPs) of the VMAT arc up to the gantry angle of position deviation event. For the rest of the treatment fraction dose, the magnitude of the position deviation that triggered the event was incorporated into the rest of the CPs dose of the VMAT arc.

Since three different position tolerance criteria were used for the monitoring in the study cohort, to avoid the ambiguity of using the larger position tolerance criteria in smaller margin plans the following approach was taken:

* All the patient plans (treated with 5 mm, 4 mm and 3 mm position tolerance) were studied with the delivered dose assessment for the margin range of 7 mm-5 mm.
* The patients treated with 4 mm and 3 mm position tolerance were studied for the delivered dose assessment of 4 mm PTV margin.
* The patients treated with 3 mm position tolerance were only used to study the delivered dose assessment of the 3 mm PTV margin.

### Analysis of delivered dose

The difference in dose delivery with and without position corrections in each PTV margin category in comparison to the respective planned dose was assessed by comparing D99 to CTV, D98 to PTV and V46 and V60 to rectum and bladder. The statistical significance of the difference of the studied DVH metrics in the scenarios of treatment with and without position corrections in comparison to the planned dose at the population level was assessed by one-way ANOVA and Tukey’s HSD test with a significance level of p<0.05.

## Results

### PTV margin

The systematic and random position errors resulting from both corrected and not corrected treatment scenarios determined based on the real-time prostate position data from the SeedTracker system are shown in **Table 1A**. The systematic and random errors were calculated for both the individual position tolerance cohort and the overall combined cohort. Similarly, the PTV margin in left-right (LR), anterior-posterior (AP) and superior-inferior (SI) directions were calculated based on van Herk’s margin recipe to cover 90% of the population with 95% of the prescription dose to CTV in both individual and overall combined cohort as shown in **Table 1B**.

**Table 1A:** The systematic and random errors of prostate position determined based on the monitoring data derived from SeedTracker system.

Direction	Corrected								Not corrected							
	Tolerance cohort								Tolerance cohort							
	5 mm		4 mm		3 mm		Overall		5 mm		4 mm		3 mm		Overall	
	Σ	σ	Σ	σ	Σ	σ	Σ	σ	Σ	σ	Σ	σ	Σ	σ	Σ	σ
LR	0.7	1.1	0.7	1.2	0.6	1.2	0.7	1.2	0.8	1.3	1	1.5	0.7	1.7	0.8	2.1
AP	0.6	1.4	0.8	1.4	0.5	1.5	0.6	1.5	0.7	2.2	1	2.2	1.3	3	1.1	2.5
SI	0.8	1.4	0.4	1.2	0.6	1.5	0.6	1.4	1.2	2.2	0.6	1.9	1.1	2.1	1	1.5

**Table 1B:** The CTV-PTV margin calculated based on the measured systematic and random errors.

Direction	Corrected				Not corrected			
	Tolerance cohort				Tolerance cohort			
	5 mm	4 mm	3 mm	All	5 mm	4 mm	3 mm	All
LR	2	2.2	1.9	2	2.3	3.2	2.4	3.1
AP	1.9	2.5	1.9	2.1	2.9	3.5	5.1	4
SI	2.5	1.4	2	2.1	4.1	2.3	3.7	3

### Plans with reduced PTV margins

**Figure 2:**
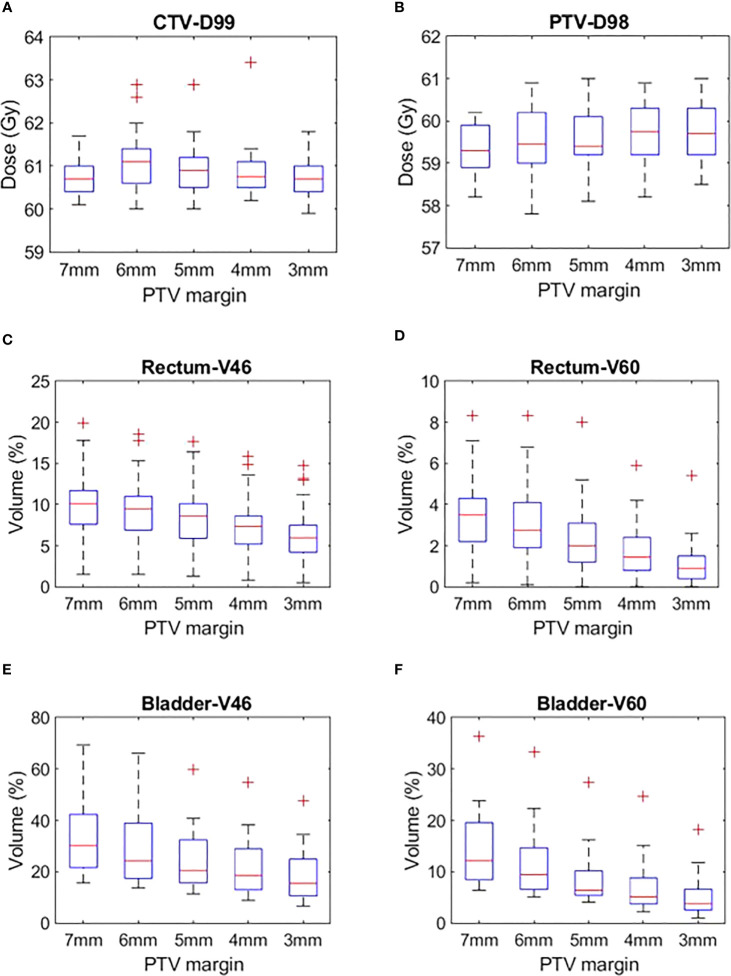
**(A)** CTV D99, **(B)** PTV D98, **(C)** Rectum V46, **(D)** Rectum V60, **(E)** Bladder V46 and **(F)** Bladder V60 plans generated with CTV-PTV margins ranging between 7 mm and 3 mm.

The target volumes and OARs DVH metrics of treatment plans generated with 7 mm, 6 mm, 5 mm, 4 mm and 3 mm PTV margins are shown in **Figure 2**. The mean ± SD of the target volume and OARs DVH metrics of the plans is shown in **Table 2**.

**Table 2:** The target volume and OARs mean DVH metrics and statistical analysis results of plans generated with CTV-PTV margin ranging between 7 mm and 3 mm.

Structure	DVH Metric		Mean ± SD of DVH metric (Gy/% volume)				
			7 mm	6 mm	5 mm	4 mm	3 mm
CTV	D99	Mean ± SD	60.8 ± 0.4	61.1 ± 0.7	60.9 ± 0.6	60.8 ± 0.3	60.6 ± 0.3
		ANOVA statistics	f = 2.2,p = 0.75				
				p=0.13	p=0.71	p=0.94	p=0.99
PTV	D95	Mean ± SD	59.9 ± 0.3	60.0 ± 0.4	60.0 ± 0.4	60.0 ± 0.3	60.1 ± 0.3
		ANOVA statistics	f = 1.94,p = 0.11				
				p=0.54	p=0.25	p=0.26	p=0.81
	D98	Mean ± SD	59.4 ± 0.6	59.6 ± 0.8	59.7 ± 0.7	59.6 ± 0.6	59.3 ± 0.5
		ANOVA statistics	f = 1.18,p = 0.32				
				p=0.91	p=0.60	p=0.47	p=0.30
Rectum	V46	Mean ± SD	10.0 ± 3.9	9.4 ± 3.8	8.6 ± 3.8	7.5 ± 3.7	6.5 ± 3.6
		ANOVA statistics	f = 4.57,p = 0.00^*^				
				p=0.86	p=0.59	p=0.08	p=0.00^*^
	V60	Mean ± SD	3.5 ± 1.8	3.0 ± 1.8	2.4 ± 1.6	1.7 ± 1.3	1.2 ± 1.1
		ANOVA statistics	f = 10.99,p = 0.00^*^				
				p=0.83	p=0.64	p=0.00^*^	p=0.00^*^
Bladder	V46	Mean ± SD	32.8 ± 13.3	28.1 ± 12.8	24.3 ± 11.3	21.3 ± 10.8	17.4 ± 9.7
		ANOVA statistics	f = 7.58,p = 0.00^*^				
				p=0.51	p=0.03^*^	p=0.00^*^	p=0.00^*^
	V60	Mean ± SD	14.5 ± 7.0	11.5 ± 6.5	8.7 ± 5.1	7.0 ± 4.7	4.9 ± 3.8
		ANOVA statistics	f = 12.42,p = 0.00^*^				
				p=0.25	p=0.00^*^	p=0.00^*^	p=0.00^*^

The statistically significant differences are denoted by ^*^.

### Target volumes dose

The CTV and PTV DVH metric goals were achieved in all the plans generated with margins ranging between 7 mm and 3 mm (**Table 2**). A one-way ANOVA revealed that there was no statistically significant difference in CTV D99 between at least two PTV margins (f-ratio = 2.17, p = 0.08). A one-way ANOVA revealed that there was no statistically significant difference in PTV D95 and D98 between at least two PTV margins (p>0.05)

### OARs dose

#### Rectum

There was a mean ± SD reduction of 1 ± 3.8% and 0.5 ± 1.8% in V46 and V60 volumes for every 1 mm reduction in PTV margin. A one-way ANOVA revealed that there was a statistically significant difference in V46 (f-ratio = 4.57, p = 0.00) and V60 (f-ratio = 10.99, p = 0.00) between at least two PTV margins. Tukey’s HSD Test for multiple comparisons found that the mean value of V46 was significantly different between 7 mm and 3 mm (p = 0.00) and 6 mm and 3 mm (p=0.01) PTV margins. Similarly, V60 was significantly different between 7 mm and 4 mm (p = 0.00), 7 mm and 3 mm (p = 0.00), 6 mm and 4 mm (p = 0.01), 6 mm and 3 mm (p=0.00) and 5 mm and 3 mm (p = 0.02) PTV margins (**Table 2**).

#### Bladder

There was a mean ± SD reduction of 4 ± 12% and 3 ± 7% V46 and V60 volumes for every 1 mm reduction in PTV margin. A one-way ANOVA revealed that there was a statistically significant difference in V46 (f-ratio = 7.58, p = 0.00) and V60 (f-ratio = 12.42, p = 0.00) between at least two PTV margins. Tukey’s HSD Test for multiple comparisons found that the mean value of V60 was significantly different between 7 mm and 5 mm (p = 0.03), 7 mm and 4 mm (0.00), 7 mm and 3 mm (0.00) and 6 mm and 3 mm (0.01) PTV margins. Similarly, V60 was significantly different between 7 mm and 5 mm (p = 0.01), 7 mm and 4 mm (p = 0.00), 7 mm and 3 mm (p = 0.00), 6 mm and 4 mm (p=0.03) and 6 mm and 3 mm (p=0.00) PTV margins (**Table 2**).

### Reduction in PTV volume

**Figure 3:**
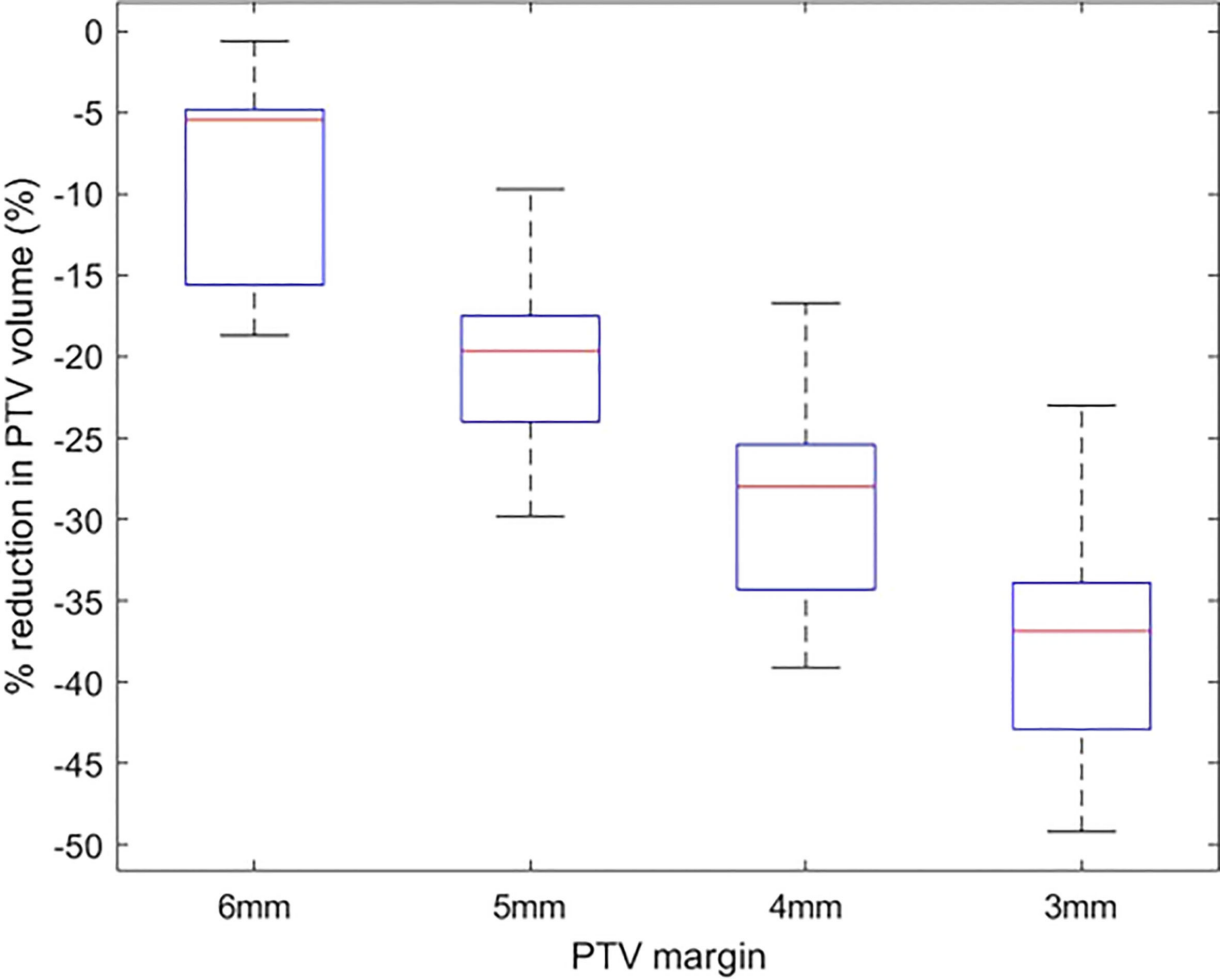
The percentage reduction in PTV volume of plans with 6 mm-3 mm PTV margin in comparison to plans with a 7 mm margin.

The change in PTV volume when the margin is reduced from 7 mm to 3 mm is shown in **Figure 3**. There is a mean ± SD reduction of 9.4 ± 5.7% for every 1 mm reduction in PTV margin.

### Delivered dose assessment

#### Target volumes dose

**Figure 4:**
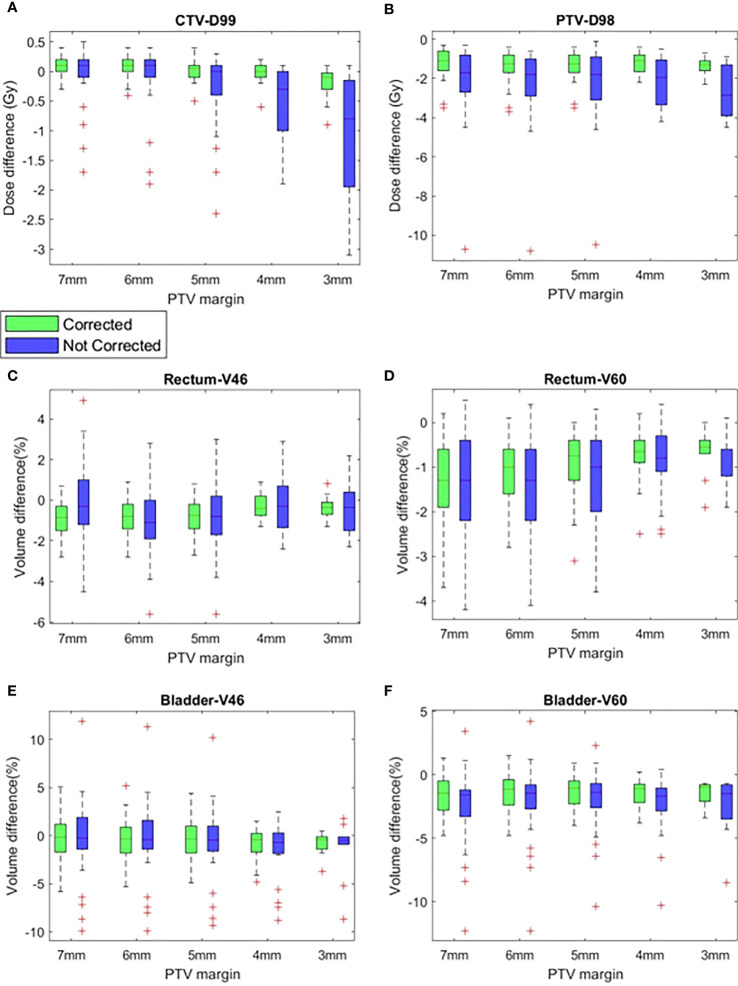
The difference between planned and delivered (corrected and not corrected scenarios) **(A)** CTV D99, **(B)** PTV D98, **(C)** Rectum V46, **(D)** Rectum V60, **(E)** Bladder V46 and **(F)** Bladder V60 of plans generated with CTV-PTV margins ranging between 7 mm and 3 mm.

**Figures 4A, B** show the difference of CTV D99 and PTV D98 between the planned and delivered with and without position corrections of the plans generated with different margins. The mean ± SD difference of CTV D99 and PTV D98 and the results of ANOVA are shown in **Table 3**. The mean difference between the planned and delivered CTV D99 was consistently small for delivery with correction compared to without corrections. A one-way ANOVA revealed that there was no statistically significant difference of CTV D99 between the planned and delivered with and without position corrections for the plans generated with 7 mm (f=0.49,p=0.61), 6 mm (f=0.46,p=0.66), and 5 mm (f=1.12,p=0.33) PTV margins. The mean CTV D99 difference with and without position correction showed the maximum difference of -0.2 ± 0.3 Gy and -1.1 ± 1.1 Gy respectively in 3 mm margin plans. A one-way ANOVA revealed that there was a statistically significant difference of CTV D99 between the planned and delivered with and without position corrections for the plans generated with 4 mm (f=0.49,p=0.61) and 3 mm (f=0.46,p=0.66) PTV margins. Tukey’s HSD Test for multiple comparisons found that the mean value of CTV D99 was significantly different between planned and delivery without corrections for 4 mm (p = 0.01) and 3 mm (p = 0.01) PTV margin plans. The mean PTV D98 difference in treatment with and without corrections showed a maximum difference of -1.4 ± 0.7 Gy and -2.7 ± 1.3 Gy in 7 mm and 5 mm PTV margin plans respectively. A one-way ANOVA revealed that there was a statistically significant difference in PTV D98 between the planned and delivered with and without position corrections for the plans generated with all PTV margins (**Table 3**). Tukey’s HSD Test for multiple comparisons found that the mean value of PTV D98 was significantly different between planned and delivered without corrections as well as between the delivery with and without corrections group. The percentage of plans meeting 95% of the prescribed dose to CTV with and without corrections is shown in **Table 4**.

**Table 3:** The difference between the planned and delivered DVH metrics of target volumes and OARs of plans generated with CTV-PTV margin ranging between 7 mm and 3 mm.

Structure	DVH metric		CTV-PTV margin									
			7 mm		6 mm		5 mm		4 mm		3 mm	
			C	NC	C	NC	C	NC	C	NC	C	NC
CTV	D99	Mean	0.1 ± 0.2	-0.1 ± 0.5	0.1 ± 0.2	-0.1 ± 0.6	0.0 ± 0.2	-0.2 ± 0.6	-0.1 ± 0.2	-0.6 ± 0.7	-0.2 ± 0.3	-1.1 ± 1.1
		ANOVA statistics	f=0.49,p=0.61		f=0.42,p=0.66		f=1.12,p=0.33		f=5.89,p=0.01		f=6.19,p=0.01	
			p=0.85	p=0.90	p=0.90	p=0.88	p=0.99	p=0.44	p=0.94	p=0.01^*^	p=0.77	p=0.01^*^
PTV	D98	Mean	-1.2 ± 0.8	-2.1 ± 2.0	-1.4 ± 0.8	-2.4 ± 2.0	-1.4 ± 0.7	-2.3 ± 2.0	-1.2 ± 0.5	-2.2 ± 1.3	-1.4 ± 0.5	-2.7 ± 1.3
		ANOVA statistics	f=13.37,p=0.00		f=14.44,p=0.00		f=15.82,p=0.00		f=17.49,p=0.00		f=19.44,p=0.00	
			p=0.01^*^	p=0.00^*^	p=0.01^*^	p=0.00^*^	p=0.00^*^	p=0.00^*^	p=0.01^*^	p=0.00^*^	p=0.01^*^	p=0.00^*^
Rectum	V46	Mean	-0.8 ± 0.9	-0.1 ± 2.1	-0.7 ± 1.0	-0.9 ± 1.8	-0.6 ± 0.9	-0.8 ± 1.7	-0.3 ± 0.7	-0.2 ± 1.4	-0.3 ± 0.6	-0.4 ± 1.4
		ANOVA statistics	f=0.39,p=0.68		f=0.44,p=0.65		f=0.37,p=0.69		f=0.02,p=0.97		f=0.03,p=0.97	
			p=0.69	p=0.99	p=0.76	p=0.65	p=0.75	p=0.72	p=0.97	p=0.98	p=0.98	p=0.97
	V60	Mean	-1.3 ± 0.9	-1.6 ± 1.4	-1.2 ± 0.8	-1.5 ± 1.2	-1.0 ± 0.8	-1.3 ± 1.1	-0.7 ± 0.6	-0.8 ± 0.8	-0.7 ± 0.6	-0.8 ± 0.7
		ANOVA statistics	f=7.20,p=0.00		f=5.69,p=0.01		f=5.92,p=0.00		f=3.99,p=0.02		f=3.59,p=0.04	
			p=0.01^*^	p=0.00^*^	p=0.04^*^	p=0.05^*^	p=0.03^*^	p=0.01^*^	p=0.08	p=0.03^*^	p=0.12	p=0.05
Bladder	V46	Mean	-0.3 ± 2.4	-0.5 ± 4.2	-0.3 ± 2.2	-0.6 ± 4.0	-0.5 ± 2.1	-0.7 ± 3.9	-0.9 ± 1.7	-1.6 ± 3.1	-0.8 ± 1.2	-1.4 ± 3.2
		ANOVA statistics	f=0.01,p=0.99		f=0.01,p=0.99		f=0.02,p=0.98		f=0.11,p=0.89		f=0.05,p=0.95	
			p=0.99	p=0.99	p=0.99	p=0.99	p=0.99	p=0.97	p=0.96	p=0.89	p=0.98	p=0.95
	V60	Mean	-1.7 ± 1.5	-2.5 ± 3.1	-1.5 ± 1.5	-2.1 ± 3.0	-1.4 ± 1.2	-2.0 ± 2.5	-1.5 ± 1.1	-2.4 ± 2.5	-1.5 ± 1.0	-2.5 ± 2.4
		ANOVA statistics	f=1.09,p=0.34		f=0.92,p=0.40		f=1.35,p=0.26		f=1.39,p=0.26		f=0.99,p=0.38	
			p=0.59	p=0.32	p=0.65	p=0.38	p=0.49	p=0.25	p=0.56	p=0.23	p=0.68	p=0.36

C- Corrected treatment scenario, NC- not corrected treatment scenario.

The statistically significant differences are denoted by ^*^.

**Table 4:** The percentage of patients receiving 95% of the prescription dose to CTV in each CTV-PTV margin category.

Margin (mm)	% of patients receiving 95% of the prescribed dose to CTV	
	Corrected	Not Corrected
7	100	100
6	100	100
5	100	93
4	100	93
3	100	87

### OARs dose

#### Rectum

**Figures 4C, D** show the difference of rectum V46 and V60 between planned and delivered with and without corrections for the plans generated with all studied margins. The delivery with position corrections consistently showed a smaller difference with planned V46 and V60 in comparison to the delivery without position corrections (**Figures 4C, D**, **Table 3**). The delivery with and without corrections showed a maximum V46 difference of -0.8 ± 0.9% and -0.9 ± 1.8% for plans with 7 mm and 6 mm PTV margins respectively. A one-way ANOVA revealed that there was no statistically significant difference of V46 between the planned and delivered with and without position corrections for the plans with all PTV margins studied (**Table 3**). The 7 mm margin plan showed the maximum V60 difference of -0.8 ± 0.9% and -0.9 ± 1.8% in delivery with and without position corrections. A one-way ANOVA revealed that there was a statistically significant difference of V60 between the planned and delivered with and without position corrections for the plans with all PTV margins studied (**Table 3**). Tukey’s HSD Test for multiple comparisons found that the mean value of V60 was significantly different between planned and delivery without corrections for the plans generated with all margins considered (**Table 3**). In delivery with position corrections, the plans with 4 mm and 3 mm PTV margins showed no statistically significant difference (p>0.05), however, the plans with 7 mm to 5 mm margins showed a significant difference (p<0.05) with the planned dose (**Table 3**).

#### Bladder

**Figures 4E, F** show the difference of bladder V46 and V60 between planned and delivered with and without corrections for the plans generated with all studied margins. The delivery with and without corrections showed a maximum V46 difference of -0.9 ± 1.7% and -1.6 ± 3.1% for plans with 4 mm PTV margin. The same for V60 was -1.5 ± 1.1% and -2.4 ± 2.5% for plans with 4 mm PTV margin (**Table 3**). A one-way ANOVA revealed that there was no statistically significant difference of V46 and V60 between the planned and delivered with and without position corrections for the plans with all PTV margins studied (**Table 3**).

## Discussion

A wide range of CTV to PTV margins are used in clinics around the world (32). The widespread implementation of CBCT based pre-treatment image guidance hoped to reduce this variation. However, the factors such as frequency of image guidance before and during treatment, type of image registration used (bone/soft tissue/implanted fiducials), the treatment technique, duration of treatment delivery time, daily position correction action levels, intrafraction motion monitoring and real-time position corrections, patient bowel/bladder preparation protocols and consideration of target delineation uncertainties contribute to the clinic’s specific margin.

The reduction of dose to OARs by modulated treatment delivery techniques (33) and accurate delivery of the same by image guidance has been shown to reduce treatment-related toxicity in prostate RT (7). The fiducials inserted in the prostate enable the ability to ensure the accurate positioning of the prostate using either the pre-treatment kV portal or CBCT image sets. The real-time prostate position monitoring enables the accurate positioning of the target volume by correcting the intrafraction position deviations resulting from internal organs motion such as peristalsis, bladder filling and bowel movement, as well as involuntary patient movements resulting from sources such as pelvis muscle relaxation and coughing. The correction for intrafraction deviations allows the reduction of the PTV margin and reducing the dose to OARs. In this study, the intrafraction prostate position deviations determined and corrected using the SeedTracker system were studied to evaluate the potential reduction of CTV-PTV margin and resulting OARs sparing. Without position correction for intrafraction motion events that exceed the action tolerance, an isotropic margin of a minimum of 4 mm is required to achieve the criteria of 90% of patients receiving 95% of the prescribed dose to CTV as per van Herk’s margin formula (29) (**Table 1B**). Studies have reported a wide range of margins to ensure CTV coverage (**Table 5**). The magnitude of the margin calculated depends on the type of treatment technique, length of treatment time, coverage criteria used for the calculation, and the number of patients included in the study. The margin calculated based on the motion data without position correction in this study agree within the range of margin reported in other studies (**Table 5**).

**Table 5:** Comparison of different studies calculated the CTV-PTV margin based on the measured intrafraction prostate motion data.

Authors	Modality	Margin (mm)			Mean treatment time (mins)	No of patients	Coverage criteria
		LR	AP	SI			
Polat et al (14)	CBCT	6	6	6	16	21	90% PP-95%PD
Beltran et al (34)	Implanted fiducial and EPI images	4.8	5.2	5.4	7	40	90% PP-95%PD
Kotte et al (35)	Implanted fiducial and EPI images	2	2	2	5-7	427	90% PP-95%PD
Skarsgard et al (36)	Implanted fiducial and EPI images	3.6	3.7	3.7	Not specified	72	95% probability of complete CTV coverage
Steiner et al (37)	Implanted fiducial and stereoscopic kV images	3.7	3.6	2.6	9.7	9	90% PP-95%PD
		2.3	6.2	3.9	15.0	3
Both et al (38)	Electromagnetic localization and tracking	3	3	3	6	24	CTV for 95% of treatment time
Wang et al (39)	Electromagnetic localization and tracking	3	3	3	6	30	Margin covering CTV for 95% of treatment time
Pang et al (40)	Clarity 4D TPUS	1.02	2.65	2.41	8	55	90% PP-95%PD
		1.84	4.63	4.29	15	
Franco et al (30)	Clarity 4D TPUS	5	5	5	7	46	95% PP-99%PD

4D TPUS- Four-dimensional transperineal Ultrasound.

90%PP-95%PD-90% of the patient population receiving 95% prescription dose.

95%PP-99%PD-95% of the patient population receiving 99% prescription dose.

Real-time monitoring with an action threshold of 4-5 mm results in the reduction of random position errors and based on the results the margin can be reduced to 2.5 mm (**Table 1B**). Reducing the position tolerance to 3 mm enables the PTV margin to be reduced to 2.0 mm. With real-time position monitoring and position corrections performed for the deviations, an isotropic margin of a minimum 2.1 mm is required to achieve the criteria of 90% of patients receiving 95% of the prescribed dose to CTV when the combined cohort of patients is considered. This is in close agreement with Badakhshi et al (16), who calculated a margin of 1.8 mm, 2.8 mm and 2.9 mm in LR, AP and SI directions is required to achieve the criteria of 90% of patients receiving 95% of the prescribed dose to CTV with the intrafraction motion corrected using Exactrac system. The position deviations that are below the action threshold results in the requirement of CTV-PTV margin in the treatment with intrafraction monitoring and corrections. Fast et al (41) experimentally demonstrated that with real-time MLC tracking to compensate for the intrafraction motion, the CTV-PTV margin can be reduced to 1 mm without adversely affecting the intended dose to CTV.

The OARs DVH metric analysis of treatment plans generated with margin ranging from 7 mm to 3 mm show that at the population level statistically significant improvement in the rectum and bladder DVH can be achieved when the margin is reduced to ≤ 5 mm (**Table 2**). Though at the individual patient level the reduction in margin would result in reduced dose to OARs, at the population level the dosimetric benefits can be achieved when the margin is reduced to ≤ 5 mm. The variation in the different magnitude of overlap between the PTV and OARs in the studied population of patients could be the possible reason for this observation. The variation in plan quality due to planners’ experience and subjectivity in manual planning could also influence it. However, all the plans in this study were generated using the Autoplanning module in the Pinnacle planning system, which was shown to generate consistently high-quality plans (42). Nevertheless, every 1 mm reduction in margin resulting in 9.4 ± 5.7% reduction in PTV volume, would result in a considerable reduction of volume of normal tissues around CTV receiving the high dose.

The delivered dose assessment that incorporates the target position during the treatment delivery shows that with the correction applied for the observed position deviations, 95% of the prescription dose to CTV is achieved in 100% when 3 mm margin was used for planning. This suggests the potential to reduce the margin without compromising the dose to CTV with real-time position monitoring and corrections.

At the population level, there is no statistically significant difference (p>0.05) in the dose delivered to CTV between the planned and treatment with and without position corrections performed for plans generated with 7-5 mm. However, the treatment of 4 mm and 3 mm plans without position corrections would result in a statistically significant difference in dose delivery to CTV in comparison to the planned dose. The Rectum V60 of the treatment with and without position correction is statistically different (p<0.05) from the planned dose for plans with 7 mm-5 mm. The residual error in the positioning and variation in V60 among the plans of the studied population could be the contributing factors for this. However, the treatment with position corrections results in a statistically insignificant difference (p>0.05) between the planned and delivered V60 to the rectum for plans with 4 mm and 3 mm margins. For bladder, the delivered V46 and V60 are no statistically different from the planned dose in both treatments with and without position correction for the plans with all studied margins. The relatively high interpatient variation of these DVH metrics that results from variations in the bladder volume and its overlap with PTV and variation resulting from the residual error could be the contributing factors for this.

Three large randomized controlled trials that assessed the efficacy of current standard-of-care radiotherapy dose regimens reported late grade 2-4 gastrointestinal toxicity in the range of 9-22% and late grade 2-4 genitourinary toxicity in the range of 7-30% (8, 43–45). Treatment-related toxicity affects patients’ Quality of Life (QoL) after treatment. This is particularly important for prostate cancer patients as they have a good prognosis and live longer. The treatment-related toxicity compounds the age-related issues and decreases the QoL of patients after treatment. A recent study that assessed 10-year post-treatment outcome data reported that about 40% of men experience long-term decrements in physical and mental QoL and life satisfaction after the treatment of prostate cancer (9). The relatively larger magnitude of the PTV margin to account for target position uncertainties increases the dose to adjacent OARs and contributes to the increased treatment-related toxicity.

The smaller treatment margins (≤3 mm) were shown to reduce the GU and GI toxicity in prostate radiotherapy. In a prospective trial, Sandler et al (46) treated 64 patients with a 3 mm PTV margin. The patient-reported outcomes were assessed using Expanded Prostate Cancer Index Composite (EPIC) questionnaire. They have concluded that a reduction in margin resulted in less RT related morbidity in comparison to the comparator patients treated with a conventional wider margin. In a similar study, Chaurasia et al (47) reported a reduction in the RT related toxicity of 31 patients with low and intermediate risk who were treated with a 2 mm PTV margin. In both these studies the intrafraction position monitoring and the corrections were performed using electromagnetic tracking and treatments were performed using a general-purpose linear accelerator. Shimizu et al (48) treated 110 prostate cancer patients with low, intermediate and high-risk categories in a real-time tumor-tracking (RTRT) radiotherapy system with a 3 mm PTV margin. In their study, the dose to prostatic urethra was restricted to V70< 10%. The acute and late adverse events were scored according to the Common Toxicity Criteria Adverse Events Version 4 (CTCAE v4.0) scale. The treatment outcome results for the median (range) follow-up period of 31.3(32-82.1)months were presented in their study and showed that they were able to achieve a very low incidence of GU and GI toxicity with the biochemical relapse-free survival equal to the highest reported in the literature.

One of the limitations of this study is that the residual rotational error after the correction is not considered in the delivered dose estimation of the plans generated with different margins. Studies have demonstrated the feasibility of reduction of PTV margin in the prostate considering both translational and rotational position corrections. Wolf et al (49) modelled the dosimetric impact of observed rotational error in prostate SBRT treatment and concluded that owing to the sphericity of the prostate they observed uncompromised dose to CTV even with 3 mm CTV-PTV margin. Another limitation of this study is that the delivered dose assessments were performed based on the planning CT dataset and the day-to-day variation of the OARs is not considered in this study. Maund et al (50) studied the delivered dose in 18 patients based on the CTV and rectum contoured on the weekly pre-treatment CBCT image data and analyzed the delivered dose by sampling the Mean DVH for these structures. Based on their study, no statistically significant difference was found between the NTCP calculated using the original planned dose and the mean rectal DVH derived from the weekly contours. Though the highlighted limitations are important to consider, the results of the present study can be used to assess the potential to reduce the margin with real-time position monitoring and corrections as these limitations are shown to have minimal impact on the delivered dose assessment. An accurate, stable and widely accessible real-time position monitoring system is essential for the wide spread adoption of margin reduction in prostate radiotherapy in the clinics. The SeedTracker position monitoring used in this study shown to accurately auto-segment the implanted seeds and isocenter position with in ±0.5mm of the ground truth data (23, 25).

Though the evidence suggests the reduction in treatment-related toxicity with the smaller magnitude of treatment margins in prostate RT (46–48), the widespread adoption of this approach is limited by the lack of real-time position monitoring capabilities in general-purpose linear accelerators. The previous studies used either the specialized treatment delivery system or additional third-party position monitoring systems which are not accessible widely. The main contribution of the present study is that the real-time prostate position data used in this study is derived using an x-ray imaging system available on the general-purpose accelerator and in-house developed position monitoring software (23–25). Using this data we demonstrated that the margin reduction can be achieved in conventional fractionation prostate RT with general-purpose linac which will reduce the OARs dose when compared to the current margin used in the clinics and this may potentially result in improved patient QoL after treatment. The advantage of using the SeedTracker system is that it eliminates the need for additional hardware systems to enable real-time position monitoring. This makes it possible to implement this method and optimize margins using existing resources, which can be cost-effective and convenient for clinical practice. By utilizing the SeedTracker system, clinics can benefit from real-time monitoring without the requirement of investing in new hardware. This approach maximizes the utilization of existing resources and simplifies the implementation of real-time position monitoring in prostate radiotherapy.

## Conclusions

The SeedTracker based real-time monitoring and position corrections resulted in improved accuracy of dose delivery in prostate RT. This study demonstrates the feasibility of reducing the margin in prostate RT with SeedTracker-based real-time monitoring without compromising the dose delivery accuracy to CTV while reducing dose delivered to critical structures of bladder and rectum.

## Data availability statement

The original contributions presented in the study are included in the article/supplementary material. Further inquiries can be directed to the corresponding author.

## Ethics statement

The studies involving human participants were reviewed and approved by Human Research Ethics Committee, South Western Sydney Local Health District. The patients/participants provided their written informed consent to participate in this study.

## Author contributions

SA developed the study concept and drafted the manuscript. KW, VD and MS contributed to data analysis and manuscript revision. All authors contributed to the article and approved the submitted version.
